# Expression of GIMAP1, a GTPase of the immunity-associated protein family, is not up-regulated in malaria

**DOI:** 10.1186/1475-2875-8-53

**Published:** 2009-04-02

**Authors:** Amy Saunders, Tracey Lamb, John Pascall, Amanda Hutchings, Carine Dion, Christine Carter, Lucy Hepburn, Jean Langhorne, Geoffrey W Butcher

**Affiliations:** 1 The Babraham Institute, Babraham Research Campus, Cambridge, CB22 3AT, UK; 2 The National Institute for Medical Research, The Ridgeway, Mill Hill, London, NW7 1AA, UK

## Abstract

**Background:**

GIMAP (GTPase of the immunity-associated protein family) proteins are a family of putative GTPases believed to be regulators of cell death in lymphomyeloid cells. GIMAP1 was the first reported member of this gene family, identified as a gene up-regulated at the RNA level in the spleens of mice infected with the malarial parasite, *Plasmodium chabaudi*.

**Methods:**

A monoclonal antibody against mouse GIMAP1 was developed and was used to analyse the expression of the endogenous protein in tissues of normal mice and in defined sub-populations of cells prepared from lymphoid tissues using flow cytometry. It was also used to assess the expression of GIMAP1 protein after infection and/or immunization of mice with *P. chabaudi*. Real-time PCR analysis was employed to measure the expression of *GIMAP1 *for comparison with the protein level analysis.

**Results:**

GIMAP1 protein expression was detected in all lineages of lymphocytes (T, B, NK), in F4/80^+ ^splenic macrophages and in some lymphoid cell lines. Additional evidence is presented suggesting that the strong expression by mature B cells of GIMAP1 and other GIMAP genes and proteins seen in mice may be a species-dependent characteristic. Unexpectedly, no increase was found in the expression of GIMAP1 in *P. chabaudi *infected mice at either the mRNA or protein level, and this remained so despite applying a number of variations to the protocol.

**Conclusion:**

The model of up-regulation of GIMAP1 in response to infection/immunization with *P. chabaudi *is not a robustly reproducible experimental system. The GIMAP1 protein is widely expressed in lymphoid cells, with an interesting increase in expression in the later stages of B cell development. Alternative approaches will be required to define the functional role of this GTPase in immune cells.

## Background

GIMAP1 (GTPase of the immunity-associated protein family 1; formerly known variously as iap38, imap38, IAN2) was the first reported member of a family of putative GTPases [1]. These are present in vertebrates, absent from bacteria, nematodes and flies but with relatives in higher plants [2-5]. Humans, rats and mice have seven or eight *GIMAP *genes clustered tightly on a single autosome. The predicted proteins encoded by these genes are similar in their amino-terminal regions, which contain a guanine nucleotide binding domain with conserved motifs, but vary significantly at their carboxy-terminal ends, which contain predicted coiled-coil regions or transmembrane (TM) domains, or both [2].

GIMAP1 was originally discovered in a differential screen of a spleen cell cDNA library made from malaria (*Plasmodium chabaudi*)-immune mice using cDNA from immune or non-immune mouse spleens [1]. In this and a later publication [6], the authors reported Northern blot comparisons of spleen mRNAs from mice either before, or seven days after, malaria infection, using both naïve and malaria-immune animals. GIMAP1 expression, which was relatively weak in naïve mouse spleen, was increased two to 30-fold post infection in various mouse strains on the C57BL/10 or C57BL/6 backgrounds and was high in spleens of immune mice both pre- and post-infection. Post-infection expression levels were particularly high in the plastic-adherent splenocyte fraction ('macrophages') and successively weaker in B and T cells [1].

GIMAP proteins are thought to be involved in the regulation of cell death. The evidence for this has come from family members other than GIMAP1, in particular GIMAP5 and GIMAP4. Diverse evidence from *in vivo *and *in vitro *systems in rat, mouse and human has indicated that GIMAP5 has anti-apoptotic properties, notably in the T lymphocyte lineage [4,7-11]. Pro-death properties, by contrast, have been ascribed to GIMAP4, from studies in both mouse and rat [4,12,13]. Consistent with the involvement of GIMAP4 and GIMAP5 in the regulation of cell survival, both proteins have been shown to be capable of interacting physically with members of the Bcl-2 family of proteins [4].

It was important to find out whether the up-regulation of GIMAP1 during malaria infection in mice was indicative of regulated apoptotic processes occurring during the immune response to this pathogen or, instead, reflected a distinct biological function for this member of the GIMAP family. The initial aim of this investigation was to generate antibodies specific for mouse GIMAP1, in order to study the expression of this GTPase at the protein level and learn more about the nature of the cells expressing it in both naïve and malaria-immune or -infected mice. A surprising outcome of this study, which made use of a novel monoclonal antibody (mAb) against mouse GIMAP1, was the failure, despite extensive efforts, to reproduce the up-regulation of GIMAP1 as reported in earlier publications [1,6].

## Methods

### Animals

C57BL/6 and C57BL/10ScSn mice for malaria experiments were bred and used at the National Institute for Medical Research; C57BL/6 mice and PVG-*RT1*^*u*^, *RT7*^*b *^rats for other experiments were bred at The Babraham Institute. LOU/C rats were obtained from Harlan UK. All animals were maintained in specific pathogen-free conditions. All husbandry and experimentation complied with UK Home Office licences and local standards in force at the respective institutions.

### Cell lines used

The mouse cell lines C1498, A20, TK-1, P815, BW5147, MTC-1, RMA, X16.C8.15 and YAC-1 were maintained in RPMI (Invitrogen) supplemented with 10% foetal calf serum (FCS), 100 units/ml penicillin and 100 μg/ml streptomycin (Invitrogen) and 25 μM β-mercaptoethanol. The mouse cell line EL4 was grown in DMEM (Invitrogen), supplemented as above. HEK293T cells were maintained in the same medium as EL4 but without the addition of β-mercaptoethanol.

### Generation of monoclonal and polyclonal antibodies

Mouse GIMAP1 was cloned into pENTR™/TEV/D-TOPO (Invitrogen) and then transferred into the Gateway pDEST15 vector using Gateway LR Clonase II Enzyme Mix (Invitrogen), in accordance with the manufacturers' instructions. In order to exclude the predicted TM domain from any expressed fusion protein product, codon 239 of the open reading frame was converted to a stop (TAA) by polymerase chain reaction (PCR) with the primers 5'-CCCAGGACCAATAAGCCAAGGTGG-3' and 5'-CCACCTTGGCTTATTGGTCCTGGG-3'. A fusion protein with glutathione-S-transferase (GST) N-terminal of mouse GIMAP1 was expressed in *Escherichia coli *Rosetta DE3 (Novagen) in inclusion bodies. These were solubilized in 6 M urea and the fusion protein was purified using sodium dodecyl sulphate-polyacrylamide gel electrophoresis (SDS-PAGE) followed by electro-elution from the gel using an electrophoretic concentrator (Isco Inc., Lincoln, Nebraska USA). A female LOU/C rat was immunized subcutaneously three times at three to four-weekly intervals with 50–100 μg of the fusion protein in Freund's adjuvant. A final, intrasplenic, boost was delivered in an aqueous vehicle. Three days later the rat was killed and spleen cells were fused with the rat Y3Ag1.2.3 plasmacytoma [14] to generate hybridomas. Hybridoma supernatants were screened by western blotting on lysates of HEK293T cells expressing myc-tagged mouse GIMAP1 (see below). The selected positive hybridoma, MAC420, secretes a rat IgG_2a _antibody. An anti-mouse GIMAP1 rabbit polyclonal antibody was custom produced by Harlan Sera-Lab (Loughborough, UK) using the same immunogen.

### Generation of epitope-tagged constructs

N-terminally myc-tagged GIMAP constructs (with the exception of mGIMAP4) were generated by cloning PCR products amplified from GIMAP cDNA, or previously cloned plasmids, into pCANmyc1 (derivative of pcDNA3 which includes an ATG and encodes an in-frame myc tag at the 5' end of the multiple cloning site; provided by Dr Simon Cook). Mouse GIMAP4 was C-terminally myc-tagged by cloning into pcDNA3.1 (-)/*myc*-HIS A (Invitrogen). The primers used are listed in Table 1.

**Table 1:** Sequences of oligonucleotide primers for protein expression of GIMAPs: cloning mouse and rat GIMAPs into pCANmyc1 or pcDNA3.1(-)/myc-HIS A

**Name**	**Forward primer**	**Reverse primer**
mGIMAP1	5'-GCATGGAATTCATGG	5'-GATGCGCGGCCGCTT
	GAGGAAGGAAGATGG-3'	ATCTGTTATTCTGGT-3'
mGIMAP1	5'-GCATGGAATTCATGG	5'-CTATCTCGAGTCCAG
aa1-aa158	GAGGAAGGAAGATGG-3'	CCAGGTCCTCTTGGC-3'
mGIMAP1	5'-CTGAGAATTCGACAC	5'-CTATCTCGAGTTGGT
aa81-aa238	CCCGGATATCTTCAG-3'	CCTGGGGGTCAGCGC-3'
mGIMAP3	5'-GCGGAATTCATGGAA	5'-GCGGCGGCCGCTTAA
	ACACTTCAGAATG-3'	ACATAAAATAAGAG-3'
mGIMAP4	5'-GCGGGATCCCATGGA	5'-GCGGGATCCCTAGTC
	AGTCCAGTGCGGTGG-3'	TTTCATAAACTGG-3'
mGIMAP5	5'-GAAACTGAATTCATGGAACAC	5'-CTTTGAGCGGCCGCACAAGGA
	CTTCAGAAGAG CACATATGG-3'	TGATGGAAGAAA TCACCC-3'
mGIMAP6	5'-GATCGGATCCAAGAT	5'-GGTCGAATTCTGGTT
	GGATTGGCTTTACAGAA-3'	ACAGTGTCTTGCTGG-3'
mGIMAP7	5'-CGTAGAATTCATGGCT	5'-CATAGCGGCCGCAAT
	GGCCAGGGAGACACT-3'	TTACCACAACTCGTCCC-3'
mGIMAP8	5'-GCGTGAATTCATGGC	5'-CGCGCTCGAGTCATT
	GACTTCATCCCACCAAG GAG-3'	TAAATGCCATAGTAATT TGG-3'
mGIMAP9	5'-CTATGGATCCATGGC	5'-GGTCGAATTCTGCTT
	TGAGCCCAGTGACAAC-3'	ATGAAAAAAGATGCC-3'
rGIMAP1	5'-CTTCGGATCCATGGG	5'-GTCAGAATTCCTGTT
	GGGAAGAAAGATGGTG-3'	ACTCTGGTCCCCAAAGG-3'

### Transient transfections

Confluent cultures of HEK293T cells were harvested and plated out at 1.5 × 10^6 ^cells per 35 mm dish. For transfections, 2 μg of plasmid DNA were diluted to 50 μl with 150 mM NaCl. Fifty microlitres of 8% (v/v) jetPEI^® ^(Autogen Bioclear) in 150 mM NaCl were added to the DNA and the mixture was incubated at room temperature for 30 min. The DNA-jetPEI^® ^complexes were added to the HEK293T cells which were then incubated for 24 hours at 37°C, 5% CO_2_. The cells were washed in phosphate buffered saline (PBS) and then lysed on ice for 10 min in 10 mM N-2-hydroxyethylpiperazine-N'-2-ethanesulphonic acid (HEPES), 150 mM NaCl, 1% (w/v) 3- [(3-cholamidopropyl) dimethylammonio]-1-propanesulphonate (CHAPS) pH7.5 containing 1:100 protease inhibitors for mammalian cells (Sigma). Insoluble cell debris was removed by centrifugation at 15,000 g at 4°C for 5 min and lysates were then diluted 1:1 with Laemmli sample buffer (Bio-Rad) containing 100 mM dithiothreitol.

### Preparation of lysates from cell lines and tissues

Cells were lysed at 10^6 ^cells per 5 μl of lysis buffer (2% NP40, 1 mM MgCl_2_, 20 mM 2-amino-2-hydroxymethyl-1,3-propanediol [Tris], 150 mM NaCl, pH 8.0, containing protease inhibitors for mammalian cells [Sigma]) and incubated on ice for 30 min. Cell debris was removed by centrifugation at 15,000 g, 4°C for 10 min. The supernatant was transferred to a fresh microfuge tube, and an equal volume of Laemmli sample buffer, containing 100 mM dithiothreitol, was added. Unless otherwise stated, lysate from 1 × 10^6 ^cells was used per lane of a polyacrylamide gel.

Tissues from male C57BL/6 mice were homogenized into lysis buffer using a Dounce homogenizer. Cell debris was removed as above. Protein concentrations were measured using the bicinchoninic acid assay (BCA) Protein Quantification kit (Pierce) and 100 μg of protein from each tissue, in 50% Laemmli sample buffer containing 100 mM dithiothreitol, was separated by reducing SDS-PAGE.

### Western blotting

Samples run on 10% polyacrylamide gels were then transferred to Immobilon P (Millipore) membrane by semi-dry blotting. Membranes were blocked overnight in 4% (w/v) milk powder in PBS containing 0.1% (v/v) Tween 20. Primary antibodies – rat mAb MAC420 anti-mGIMAP1, rat mAb MAC417 anti-mouse/rat GIMAP4 [11], rabbit anti-mGIMAP1 polyclonal serum, rabbit anti-rat GIMAP8 polyclonal serum [15], mouse mAb 9E10 anti-myc tag (prepared in-house) and mouse mAb AC-15 anti-β-actin (ascitic fluid, Sigma) – were incubated on the membrane in the blocking solution for 1 hr, before repeated washing with PBS containing 0.1% (v/v) Tween 20. Secondary antibodies (horseradish peroxidase [HRP]-conjugated goat anti-rat IgG (Jackson), HRP-goat anti-rabbit IgG (DAKO Cytomation) or HRP-goat anti-mouse IgG (Sigma A4416)) were similarly applied. The membranes were washed as previously described and developed with the West Pico SuperSignal Reagent (Pierce). Where applied, densitometirc quantification of Western blot bands was performed using a Labworks 4 Bioimaging System (UVP).

### Purification of cell populations by fluorescence-activated cell sorting (FACS)

Single cell suspensions were prepared from mouse spleen, thymus, bone marrow or lymph nodes and, if required, red blood cells were lysed using 3 mM Tris, 140 mM NH_4_Cl, pH 7.2 for 10 min at 37°C. Cells were washed with PBS containing 5% FCS and then incubated with primary antibodies for 30 min on ice. After further washing with PBS-5% FCS, they were incubated with secondary antibodies for 30 min on ice in the dark. The cells were finally washed again and passed through a 40 μm filter before being sorted using a FACSAria^® ^(BD Biosciences). MAbs against the following mouse cell surface markers were used: B220 (RA3-6B2, BD Pharmingen, biotin- or allophycocyanin-conjugated), CD4 (YTS177 or YTS191.1, gifts from Dr Jenny Phillips; biotin-conjugated in-house), F4/80 antigen (Serotec, biotin-conjugated); CD3 (KT3 a gift from Dr Denis Alexander, fluorescein isothiocyanate-conjugated), CD8 (KT15 or YTS169), IgM^b ^(AF6-78, BD Pharmingen, fluorescein isothiocyanate-conjugated), NK1.1 (PK136, BD Pharmingen, R-phycoerythrin-conjugated), IgD^b ^(217-170, BD Pharmingen, R-phycoerythrin-conjugated), CD19 (1D3, BD Pharmingen, R-phycoerythrin-conjugated), CD25 (PC61, BD Pharmingen, R-phycoerythrin- or allophycocyanin-conjugated). Cy5.5-conjugated streptavidin (BD Biosciences) was used as a secondary reagent to detect the biotinylated antibodies. The cell populations obtained had purities of between 90–100% by FACS analysis.

### Infection with Plasmodium

The clones *AS *and *CB *of *Plasmodium chabaudi chabaudi *[16] were maintained as frozen stocks and passaged in mice as described previously [17]. For experiments, mice were injected intra-peritoneally with 10^5 ^or 10^6 ^infected red blood cells (iRBC) diluted in 100 ml Krebs' saline. Infections were monitored by microscopic examination of Giemsa-stained thin blood films as described [18].

### Erythrocyte ghost preparations

Naïve and infected C57BL/10ScSn mice were exsanguinated and their red blood cells were separated by diluting the blood with a four-fold excess of RPMI supplemented with 1 mM HEPES followed by centrifugation on a Percoll^® ^cushion (7.4 ml isotonic Percoll^® ^mixed with 2.6 ml RPMI-1 mM HEPES) at 400 g for 11 min at 15°C with no brake. The parasitized red blood cells were removed from the medium-Percoll^® ^interface and were washed with Iscove's Complete Medium. Ghosts were prepared in a manner similar to that described by Wunderlich and colleagues [19]. Parasitized red blood cells were concentrated in Krebs' saline supplemented with 3% FCS. An equal volume of Glycerol Buffer (10% glycerol, 5% FCS in PBS) was added and the cells were incubated at room temperature for 1.5 min. A 4-fold volume of RPMI-5% FCS was added and the parasites and ghosts were separated on a 1.01–1.02 g/ml one-step Percoll^® ^gradient which was centrifuged at 5000 g for 30 min. Ghosts were recovered from the top of the gradient, washed with RPMI-5% FCS and centrifuged again. They were resuspended in phosphate buffer (5 mM NaH_2_PO_4_/NaHPO_4_, pH 8.5), spun down and washed again in RPMI-5% FCS, and finally resuspended in PBS. Erythrocyte ghosts from 1 × 10^7 ^red blood cells were used per mouse for subcutaneous immunization in complete Freund's adjuvant. Seven days post-immunization, some of the mice were infected with 10^6 ^parasitized red blood cells as described above. Nine weeks after this infection the mice were infected again, and seven days later mice were taken as "immune".

### Preparation of cDNA

Total RNA was extracted from cells using TRIZOL reagent (Invitrogen) and was used to generate first-strand cDNA using SuperScript III RNase H^- ^reverse transcriptase (Invitrogen).

### Real-time PCR

Real-time PCR reactions were carried out as described previously, using cirhin and 6-phosphofructokinase C as control genes [15]. The primers used are listed in Table 2.

**Table 2:** Sequences of oligonucleotide primers for Real Time PCR analysis of *GIMAP*s

**Name**	**Accession #**	**Forward primer**	**Reverse primer**
*Internal control genes*			
6PFKc	Y19008	5'-GAGTGGAGC	5'-CACGTTGAGGTA
		GGACTTCTGGA-3'	GGAATACTTCTGC-3'
Cirhin	BC027399	5'-ATTACGATGC	5'-CCACAGTTCCAA
		TGCTCTCCGAA-3'	GTGATGCG-3'
*GIMAP genes*			
GIMAP1	NM_175860	5'-TTCAGGGACT	5'-TTCCTCATCTCT
		GTCAGCAAGAA-3'	TGCCATCTTC-3'
GIMAP3	AF337052	5'-GGACTGGCTG	5'-AGGATCCTCAGT
		GACCAGAGACT-3'	GGCCTTGAC-3'
GIMAP4	NM_174990	5'-GAAAGTTCAA	5'-CCCCAAGGATAC
		GGAGCAGCCATGA-3'	TGTTCCCTGTT-3'
GIMAP5	NM_175035	5'-GGAACACCTT	5'-TCCTCAGGCAGC
		CAGAAGAGCACAT-3'	TAGATTCTTGT-3'
GIMAP6	NM_153175	5'-GTGAGCACAG	5'-ACCAGAAGGAGC
		ACCTGAGAAGAATC-3'	TGCAGTCTCT-3'
GIMAP7	NM_174960	5'-AGAGATCCTGAACT	5'-CATGTCAAGGCA
		CTCATTAACTGTTG-3'	AGCTGACTCTA-3'
GIMAP8	AB178029	5'-AGCCTTGCGT	5'-TCCCCAGGATAG
		CATCAGAGAGAAA-3'	TGTTCCCAGTT-3'
GIMAP9	NM_146167	5'-CCTGGTGCAG	5'-CTTAACGTGGAA
		TGCTCTCCGAA-3'	GCTCTGGAAT-3'

## Results

### A monoclonal antibody specific for mouse GIMAP1

**Figure 1:**
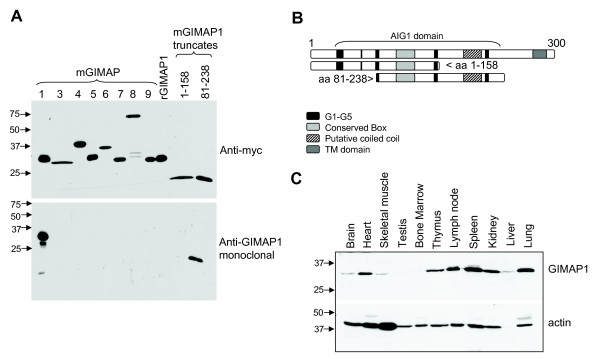
**An anti-mouse GIMAP1 monoclonal antibody was produced that is specific and can detect endogenous mouse GIMAP1**. *A*. N-terminally myc-tagged GIMAP constructs were transiently transfected into HEK293T cells. The lysates were Western blotted with either an anti-myc antibody 9E10 (*upper panel*) or the anti-GIMAP1 mAb MAC420 (*lower panel*). *B*. The domain structure of mouse GIMAP1 and the two truncates used in *A*. G1-G5 are the GIMAP versions of the canonical GTP binding motifs, TM indicates the putative transmembrane domain. *C*. Tissues were removed from a C57BL/6 mouse, homogenised in lysis buffer and 100 μg of protein from each sample was Western blotted with the anti-GIMAP1 mAb (MAC420) or anti-β-actin (AC-15). Estimated molecular weights are in kDa.

A rat mAb, MAC420, was generated against a recombinant mouse GIMAP1 fusion protein. The specificity of this antibody was assessed by western blots of lysates from a series of transiently-transfected HEK293T cells expressing different myc-tagged GIMAP constructs, including a full set of the mouse GIMAP family (Figure 1A). MAC420 detected full-length mouse GIMAP1, but not the rat homologue. It reacted selectively with GIMAP1, failing to detect any of the other mouse GIMAPs. The antibody reacted with a myc-tagged construct containing predicted amino acids 81–238 but not with a construct containing predicted amino acids 1–158 of mGIMAP1, suggesting that the epitope for this mAb lies between amino acids 158 and 238 (Figure 1B).

A rabbit antiserum raised against mGIMAP1 was also tested on the lysates from transiently transfected HEK293T cells. This polyclonal reagent detected full-length mouse GIMAP1 and both of the truncated constructs described above.

### GIMAP1 protein in normal mouse tissues

Blots of homogenized tissues from male C57BL/6 mice were probed using the MAC420 antibody. Figure 1C shows that MAC420 detected a protein of the size expected for GIMAP1 in tissues of the immune system, with high levels of expression being found in spleen, lymph node and thymus. GIMAP1 expression was also found at high levels in heart, lung and kidney.

### Expression of GIMAP1 in cells from spleen, thymus and bone marrow

**Figure 2:**
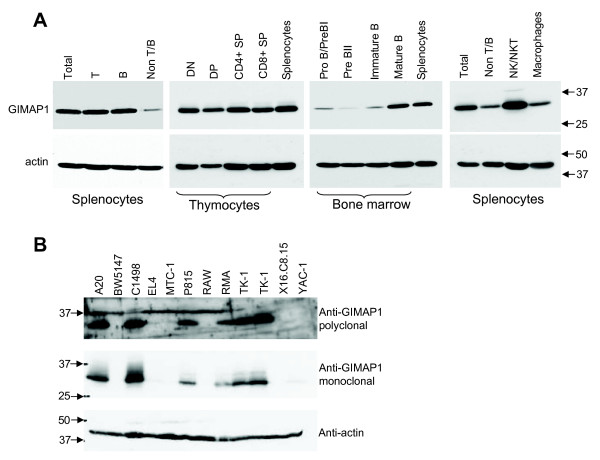
**GIMAP1 is expressed endogenously in various immune cell populations and in cell lines**. Cells were purified from C57BL/6 mice by FACS sorting with markers as follows: T cells (CD3+), B cells (B220+), non T/B cells (CD3- B220-), DN thymocytes (CD4- CD8-), DP thymocytes (CD4+ CD8+), CD4+ SP thymocytes (CD4+ CD8-), CD8+ SP thymocytes (CD4- CD8+), Pro B/Pre BI cells (B220+ CD25- IgD- IgM-), PreBII cells (B220+ CD25+ IgD- IgM-), immature B cells (B220+ CD25- IgD- IgM+), mature B cells (B220+ CD25- IgD+ IgM+), NK cells (NK1.1+), macrophages (F4/80+). Cell lysates were Western blotted with MAC 420 anti-GIMAP1 or anti-β actin. *B*. Lysates from the indicated mouse lymphomyeloid cell lines were Western blotted with MAC 420 anti-GIMAP1, a rabbit anti-GIMAP1 polyclonal antibody or anti-β actin.

To elucidate which cell types were expressing GIMAP1 in immune tissues, cell suspensions prepared from male C57BL/6 mice were separated by FACS and lysates were gel-separated and western-blotted using MAC420 (Figure 2A). GIMAP1 was found predominantly in the T (CD3^+^) and B (B220^+^) cells of the spleen and to a lesser extent in the non-T/non-B (CD3^- ^B220^-^) cell fraction.

Thymocytes were separated into CD4^-^CD8^- ^double-negative (DN), CD4^+^CD8^+ ^double-positive (DP) and CD4^+^CD8^- ^or CD4^-^CD8^+ ^single-positive (SP) populations. GIMAP1 was detectable in all of these thymocyte populations at a similar level, which was a level comparable to that found in splenocytes.

B cells from bone marrow were separated on the basis of CD25, B220, IgD and IgM expression into pro-B and pre-BI cells (CD25^- ^B220^+ ^IgD^- ^IgM^-^), pre-BII cells (CD25^+ ^B220^+ ^IgD^- ^IgM^-^), immature B cells (CD25^- ^B220^+ ^IgD^- ^IgM^+^) and mature B cells (CD25^- ^B220^+ ^IgD^+ ^IgM^+^). GIMAP1 expression was highest in the mature B cells and at a substantially lower level in the less mature subsets.

Closer investigation of the non-T, non-B fraction of splenocytes revealed that GIMAP1 was expressed at a high level in NK/NKT (NK1.1^+^) cells, while there was only a relatively low level of GIMAP1 in splenic macrophages (F4/80^+^). This contrasts with the high level of GIMAP1 mRNA expression attributed to macrophages in malaria-immune spleen by Krücken and colleagues [1]. GIMAP1 was also found to be expressed in bone marrow-derived, GM-CSF cultured, dendritic cells.

### GIMAP1 is expressed endogenously in some lymphomyeloid cell lines

Lysates from a variety of cultured lymphomyeloid mouse cell lines were analysed by western blotting using the polyclonal rabbit antiserum against mouse GIMAP1 and with mAb MAC420 (Figure 2B). GIMAP1 was not expressed in all of the cell lines tested but was found at significant levels in C1498, an NKT cell line [20], TK-1 (thymoma) and A20 cells (B cell lymphoma), and at a lower level in P815 cells (mastocytoma). The two serological reagents gave concordant results.

### GIMAP1 was not up-regulated in mice infected with two strains of Plasmodium chabaudi: AS and CB

**Figure 3:**
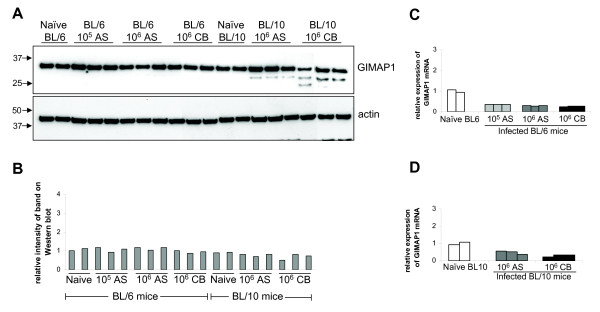
**GIMAP1 is not up-regulated in mice infected with *Plasmodium chabaudi AS *or *CB***. C57BL/6 and C57BL/10 mice were infected with *Plasmodium chabaudi AS *or *CB *(a more virulent strain of the parasite). Seven days post-infection mice were culled and the levels of GIMAP1 protein and mRNA were measured. *A*. GIMAP1 protein levels were measured by Western blotting of spleen cell lysates from naïve or infected mice using the GIMAP1 monoclonal antibody (MAC420). Anti-β actin served as a loading control. *B*. The intensities of GIMAP1 bands in the Western blot in *A *were quantified by densitometry and plotted relative to the average intensity of the bands for the C57BL/6 naïve mice. *C*. and *D*. RNA was extracted from the spleen of each mouse and Real-time PCR was used to measure the levels of GIMAP1 relative to the average of two naïve mice, which was set to a value of 1. Bars represent individual mice.

It had previously been reported that GIMAP1 expression, measured at the mRNA level, was elevated in the spleens of mice with malaria [1,6]. In order to learn more about the cellular details of this rise in expression, for which the mAb MAC420 could be a useful novel tool, C57BL/6 mice were infected with *P. chabaudi AS*, or the more virulent *CB *strain, for seven days and then killed. Spleens were taken and mRNA and protein levels were examined. Surprisingly, no up-regulation of GIMAP1 expression was seen at either the mRNA or protein level, irrespective of the virulence of the parasite or number of parasites used for infection (Figure 3). GIMAP1 was also not up-regulated in either male or female mice on days 4, 7 or 12 of infection. The level of GIMAP1 in other tissues was also examined and no significant up-regulation was found in heart, brain or liver.

### Mice made immune to malaria did not show elevated GIMAP1 levels

**Figure 4:**
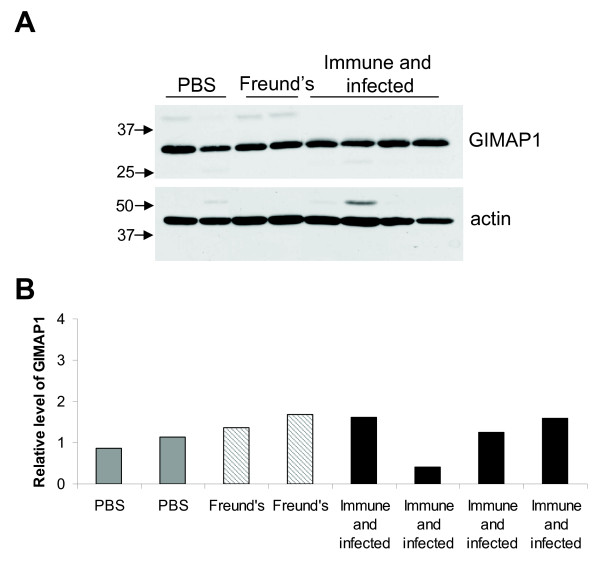
**GIMAP1 is not up-regulated in C57BL/10 mice immune to *Plasmodium chabaudi *infection**. C57BL/10 mice were immunised with erythrocyte ghosts from infected mice in Freund's adjuvant. Seven days later the mice were infected with *Plasmodium chabaudi AS*. After nine weeks the mice were infected a second time. Seven days post-infection the mice were culled and analysed for GIMAP1 expression (immune and infected mice). *A*. Spleen lysates were Western blotted with the anti-GIMAP1 mAb MAC420 or anti-β actin. *B*. RNA was extracted from spleens, cDNA was synthesized and real-time PCR was used to measure relative GIMAP1 levels. 'PBS' denotes mice that were given PBS alone instead of erythrocyte ghosts and were not infected; 'Freund's' denotes mice that were given Freund's adjuvant without erythrocyte ghosts and were not infected. Levels of GIMAP1 were measured relative to the levels of two control genes, cirhin and 6-phosphofructokinase.

**Figure 5:**
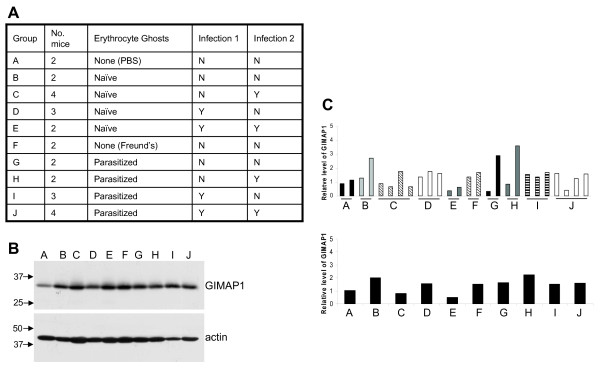
**GIMAP1 was not up-regulated in mice immune to malaria infection or in mice which had received other combinations of the treatments to make the mice immune**. *A*. Details of the treatment of each group of mice: mice were immunised with erythrocyte ghosts from naïve or parasitised mice in Freund's adjuvant, or were given Freund's alone, or PBS. Seven days later mice were infected with 10^6 ^*Plasmodium chabaudi AS *parasites (Infection 1, Y), or were given Krebs' saline alone (N). After nine weeks mice were infected with 10^6 ^*Plasmodium chabaudi AS *parasites (Infection 2, Y), or were given Krebs' saline alone (N). On day 7 post infection mice were culled and analysed for GIMAP1 expression. *B*. Representative spleen lysates were separated by SDS-PAGE and Western blotted with the anti-GIMAP1 mAb MAC420 and anti-β-actin. *C*. The expression of GIMAP1 mRNA in spleen samples was measured by real-time PCR, arbitrarily setting the mean of the two Group A samples at 1. Mean values for GIMAP1 expression levels for each group of mice are shown in the lower histogram. Mouse group designations are given beneath the histograms.

To examine whether mice that were "immune" to malaria had elevated GIMAP1 levels, as had been previously reported [1], C57BL/10 mice were similarly immunized with erythrocyte ghosts from infected mice. After seven days, the mice were infected with *P. chabaudi AS *and, nine weeks post infection, re-infected with *P. chabaudi AS*. On day 7 after the second infection, the mice were taken as "immune mice" following the published protocol [1]. At the protein level, GIMAP1 expression in spleens from "immune" mice was the same as that observed in mice given PBS only or Freund's adjuvant only (Figure 4). Various other combinations of erythrocyte ghost treatment from infected or naïve mice, and infection or immunization with vehicle were tried and no treatment was found to significantly up-regulate GIMAP1 (Figure 5).

### Species variation in GIMAP expression by B cells

**Figure 6:**
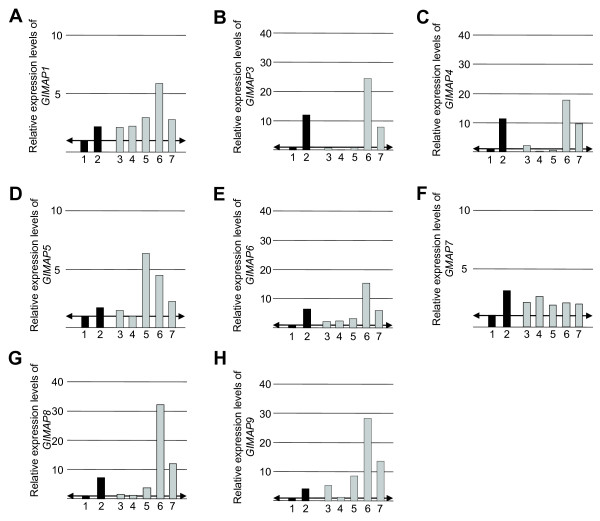
**Relative expression of *GIMAP *genes in bone marrow B cell subpopulations from C57BL/6 mice**. Real-time PCR was used to compare *GIMAP *gene expression in bone marrow B cell subpopulations with that in lymph node B cells, lymph node CD4+ T cells and DN thymocytes. (T cells [black square]; B cells: [grey square]). Bar 1: DN (CD4-CD8-) thymocytes (reference); bar 2: lymph node CD4+CD8- T cells (control, strong expression); bar 3: bone marrow pro-B and pre-BI; bar 4: bone marrow pre-BII; bar 5: bone marrow immature B; bar 6: bone marrow mature B cells and bar 7: LN CD19+ B cells. (A-H) Expression levels of, respectively, *GIMAP1, GIMAP3, GIMAP4, GIMAP5, GIMAP6, GIMAP7, GIMAP8 *and *GIMAP9*. ↔ Reference level (set at 1) corresponding to the DN population. The data are representative of three separate experiments conducted in triplicate. Cell populations were purified by FACS using the cell surface markers indicated (or see Fig.2 legend for cell surface markers for bone marrow B cell subsets).

**Figure 7:**
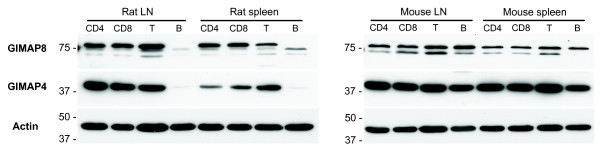
**Protein expression of GIMAP4 and GIMAP8 in FACS-sorted populations of mouse and rat lymphocytes**. Expression of GIMAP4 and GIMAP8 by mouse and rat lymphocytes was compared using the anti-GIMAP4 mAb MAC417 and rabbit anti-rat GIMAP8 polyclonal antiserum (both being species-crossreactive reagents) by Western blotting. Lymphocytes from lymph node and spleen were flow-sorted into T and B cells and CD4+ and CD8+ T cell populations (95 – 99.8% purity). The equivalent of 1 × 10^6 ^lysed cells was loaded per lane. The blots were stripped and stained with monoclonal anti-actin antibody as a loading control.

The results shown in Figure 2 revealed a difference in GIMAP1 expression in the T and B cell lineages. Whereas GIMAP1 protein was expressed relatively highly by cells at all of the stages in T cell development from DN thymocytes to mature splenic T cells, cells in the early stages of B cell development were found to express it only weakly, with a significant up-regulation between the immature and mature B cell stages. In order to find out whether this was a unique feature of GIMAP1 expression, Real Time-PCR analysis of the expression of all the *GIMAP *gene family members in B cell development was conducted. The results showed (Figure 6) an up-regulation in the expression of all the *GIMAP *genes during B cell maturation, with the exception of *GIMAP7*. It was also notable that the levels of GIMAP mRNA detected in mature B cells exceeded those for CD4^+ ^lymph node T cells for the same family members. These results stood in striking contrast to the results of an analysis of *GIMAP *gene expression in rats conducted and published previously [15]. In that study, B cell expression of each of the *GIMAP *genes was lower than that by lymph node T cells. It was of interest to determine whether this expression difference was also seen at the protein level. Unfortunately, no antibody suitable for comparing GIMAP1 protein expression in mouse and rat lymphocytes was available (the crossreactivity of our rabbit polyclonal anti-mGIMAP1 with rat was insufficiently good). It was possible, however, to compare the expression of mouse and rat GIMAP4 and GIMAP8 using reagents developed previously, *i.e*. the crossreactive mAb MAC417 against GIMAP4 [12] and a polyclonal rabbit antiserum against rat GIMAP8 [15]. The results (Figure 7) showed that the expression of GIMAP4 and GIMAP8 protein is substantially higher in mouse than in rat spleen or lymph node B cells. The protein expression in mouse B cells appeared to approach the levels found in T cells of both species.

## Discussion

Recent reports on the functional and biochemical properties of GIMAP4 and GIMAP5 in mice and rats [4,12,13] have led to the hypothesis that members of this family are critical regulators of T cell survival during development, selection and homeostasis [21]. The evidence for a requirement for GIMAP5 in T cell generation and survival is compelling, coming as it does from independent experimental systems. These are, respectively, the analysis of the rat *lymphopenia *mutation present in the type I diabetic BB rat, which has been shown to be due to a premature stop in the *GIMAP5 *open reading frame [22,23], the development of a mouse strain bearing a targeted deletion of the mouse *GIMAP5 *gene [11] and the use of mouse reaggregate foetal thymic organ culture to examine *GIMAP *gene function by shRNA knockdown [4]. Similarly, evidence from both a knockout mouse strain and a hypomorphic rat variant strain has implicated GIMAP4 in the acceleration of cell death processes in mature T lymphocytes [12,13].

The tight genomic clustering of the *GIMAP *genes within as little as 100 kb of autosomal DNA prompts the idea that they are coordinately regulated and participating in related functional activities. The data on GIMAP4, GIMAP5 and GIMAP3 [a very close relative of GIMAP5 so far identified only in mice [4,24]] and their roles in T cell development lend support to this proposal but corroborative evidence with respect to the other members of the GIMAP family is so far lacking. In the case of GIMAP1, however, the published data on its induction in a mouse model of malaria infection were consistent with a possible role in lymphoid (and/or myeloid) cell responses/homeostasis [1,6]. A re-examination of these findings was undertaken, making use of novel serological reagents to assess GIMAP1 at the protein level. Using the specific mAb MAC 420, GIMAP1 was shown to be highly expressed in lymphoid tissues, lung, kidney and heart (Figure 1C), which is reasonably consistent with published results and on-line data resources [4,25]. Unlike that of GIMAP4 [15,26], expression of GIMAP1 in the stages of thymic T cell development showed little variation (Figure 2A), a result consistent with previously published real-time PCR analysis [4,13]. By contrast, immature stages in B cell development expressed less GIMAP1 than mature B cells (Figure 2A). Purification of spleen cell subsets revealed similar levels of GIMAP1 expression in T and B lymphocytes, modest expression in the F4/80^+ ^macrophage subset and highest expression (on a per cell basis) in NK/NKT cells. The immunohistological appearance of normal spleen showed positive staining in both T and B cell areas, in agreement with the blotting data from separated cells. A number of mouse cell lines expressed GIMAP1: these came from various lineages – T, B, NKT and mast cell – consistent with the wide distribution seen in lymphoid tissues (Fig. 2B). The relatively high expression of GIMAP1 by mature mouse B cells was investigated further since it contrasted with gene expression data obtained for rats [15]. Results obtained using both RT-PCR and western blotting approaches (Figures 6 and 7) suggest that there may be a concerted up-regulation in the expression of most if not all the GIMAPs in mature mouse B cells. This appears to be a species-specific phenomenon since it was not seen in rats, and reference to on-line gene expression databases suggests that it is also not a feature of the expression of GIMAPs in humans [25].

Surprisingly, and despite extensive efforts, we were unable demonstrate up-regulation of GIMAP1 in an experimental malaria infection as had been described previously [1,6]. This was the case not only for a primary response to infection (Figure 3) but also for mice made "immune" to malaria following the protocol described in the original papers (Figure 4) and for two differently virulent strains of the parasite. Indeed, GIMAP1 expression remained stubbornly constant despite a variety of treatment schedules that were tried out (Figure 6). It has to remain a possibility that an unidentified and uncontrolled variable in the conduct of the experiments was responsible for this. In this regard it is worthy of note that a recently published dissertation originating from the laboratory in which the original findings were obtained reports a similar failure to observe GIMAP1 induction [27]. Chemical or microbiological contamination of the *P. chabaudi *stocks might have led to the original results but we have failed to up-regulate GIMAP1 expression in splenocytes *in vitro *using a range of Toll-like receptor ligands or cytokines.

Our studies of GIMAP1 have forced a reconsideration of the conclusions drawn previously about the role that this gene may be playing in a favoured mouse model of malaria. Given the evidence for substantially coordinated regulation of *GIMAP *gene expression, it seems likely that, in common with the better-studied family members such as GIMAP5, GIMAP1 will be shown to participate in the regulation of apoptosis in lymphomyeloid cells. Data consistent with this possibility have come from a study in which cell death was induced in a myeloid leukemic cell line, LTR6, carrying p53 under the control of a temperature-sensitive promoter [28]. When p53 expression was induced by a temperature shift to 32°C, *GIMAP1 *was one of several genes that showed increased expression at the RNA level. After 24 hrs, a 6-fold increase in GIMAP1 mRNA was observed by microarray analysis. Our own attempts to establish a survival function for GIMAP1 via siRNA knockdown of its expression have been inconclusive so far. A maximum of only 60% knockdown of protein expression could be achieved in a cell line, and this had no effect on the cells' susceptibility to a number of pro-apoptotic insults (data not shown). Of course, a critical survival requirement for GIMAP1 might doom to failure a simple knockdown approach, since cells in which this protein has been efficiently forced down might not survive the selection procedure. It is possible that germline modification methods in mice may be required to establish the function of GIMAP1.

## Conclusion

The reported up-regulation of GIMAP1 expression in C57BL mouse spleen in response to *P. chabaudi *[1,6] is not a robust phenomenon. Defined conditions in which the effect could be reproduced were sought without success. Unfortunately, this experimental malaria system does not appear to be a promising one for the further investigation of *GIMAP *gene function. It also seems unlikely that the study of this gene family holds particular promise for our understanding of the resistance to malaria characteristic of the C57BL strains of mice [29]. The monoclonal antibody MAC420 should prove to be a useful reagent for the further characterization of the role of this GTPase in immune cells.

## Competing interests

The authors declare that they have no competing interests.

## Authors' contributions

AS generated the immunogen for the production of the anti-GIMAP1 hybridoma, analysed the expression of GIMAP1 protein, conducted malaria infections and QPCR on malaria-infected samples and drafted the manuscript (with GB & JP). TL conducted malaria infections, and designed the malaria experiments (with JL). JP drafted and revised the manuscript (with AS, GB). AH made the anti-GIMAP1 hybridoma (with AS). CD validated the QPCR primers and controls and measured GIMAP mRNA levels in lymphocyte subsets. LH and CC purified lymphocyte subsets from rats and mice and western blotted for GIMAP4 and GIMAP8. LH also generated the immunogen for, and validated, the anti-GIMAP8 antiserum. JL designed the malaria experiments (with TL). GB conceived the project and drafted and revised the manuscript (with AS, JP).
